# Study of Short-Term Outcome of Surgically Managed Displaced Pediatric Radial Neck Fractures: A Case Series

**DOI:** 10.7759/cureus.30947

**Published:** 2022-10-31

**Authors:** Sreekanth Kashayi-Chowdojirao, Sridhar Chirla, Srikanth Eppakayala, Safia Sultana, Maheshwar Lakkireddy

**Affiliations:** 1 Orthopaedics, Nizam’s Institute of Medical Sciences (NIMS), Hyderabad, IND; 2 Orthopaedics, Osmania Medical College, Hyderabad, IND; 3 Orthopaedics, All India Institute of Medical Sciences (AIIMS), Hyderabad, IND; 4 Orthopaedics, Nizams Institute of Medical Sciences (NIMS), Hyderabad, IND

**Keywords:** radial neck fractures, metaizeau technique, judet classification, fall on out streched hand, posterior interosseous nerve palsy, stiff elbow, paediatric elbow injuries

## Abstract

Introduction: Pediatric radial neck fractures are relatively rare elbow injuries commonly seen in children between eight to 12 years of age. Judet type III and Judet type IV radial neck fractures require surgical intervention for optimal functional outcomes. The present study evaluates the functional results of Judet type III and IV radial neck fractures operated at a single center.

Materials and methods: This is a retrospective study conducted by using medical records of nine patients who had displaced radial neck fractures (Judet type III and type IV) treated at our institute which is a tertiary trauma care center between January 2012 and December 2021. Patients were assessed for functional outcome by Mayo elbow performance score (MEPS), the Tibone and Stoltz functional criteria, and for complications with the average follow-up of four years (range: six months to seven years).

Results: The mean age of the patients was 9.14 ± 2.2 years (range: four to 11 years). Seven (77.8%) patients were males and two (22.2%) patients were females. The right side was the most commonly injured side (right at 67%, left at 33%). Five (55%) cases were of Judet type III and four (45%) cases were of Judet type IV. Three cases treated with closed reduction and intramedullary nailing by the Metaizeau technique had excellent functional results. Among two patients treated with percutaneous pin leverage and intramedullary nailing by the Metaizeau technique, one patient had an excellent outcome, and the other had a good outcome. Among four cases treated with open reduction and K-wire fixation, two patients had good outcomes, one patient had a fair outcome, and one patient had a poor outcome.

Conclusion: The majority of moderately to severely displaced pediatric radial neck fractures which need intervention can be managed by the closed reduction technique of Metaizeau with or without pin leverage with excellent to good functional outcomes at short-term follow-up. Some cases need open reduction which also has good to fair outcomes. Initial trauma and associated injuries seem to play a role in the outcome rather than the treatment method per se. However, a larger sample size and longer follow-up are needed for comparisons and for arriving at better and definitive conclusions.

## Introduction

Elbow injuries are common in children but radial neck fractures are relatively rare (5% to 10%) in pediatric elbow fractures [1,2]. Children between eight to 12 years of age are frequently prone to radial neck fractures. The most common mechanism of injury is a fall on the outstretched arm with the forearm in supination and an associated valgus thrust causing compression on the radiocapitellar joint [3]. Radial neck fractures in children are classified by Judet et al. into four types based on the displacement/angulation of the radial head [4].

The key factors that decide the treatment plan are fracture displacement, fracture angulation, and the age of the patient. Less than 30 degrees of angulation at the fracture site can be accepted in children as remodeling potential corrects the alignment as the child grows and hence radial neck fractures with angulation less than 30 degrees are treated with closed reduction and immobilization in a plaster cast [5]. Displaced radial neck fractures with angulation greater than 30 degrees (Judet type III and type IV) should be treated surgically [6]. If the child is very young (< 10 years) up to 45-degree angulation can be managed conservatively.

The available treatment options for Judet type III and type IV radial neck fracture are a) closed reduction and immobilization in a plaster cast, b) percutaneous pin leverage method, c) elastic stable intramedullary nailing, d) open reduction with or without internal fixation [7-12]. The proximal radial epiphysis has retrograde blood supply as blood vessels enter at metaphysis and supply proximally to the radial epiphysis [13]. Percutaneous pin reduction and open reduction with or without internal fixation may further damage the blood supply and capsule of the elbow joint leading to complications like avascular necrosis of proximal radial epiphysis and elbow stiffness. When feasible, closed reduction with gentle manipulation and intramedullary nailing (as first described by Metaizeau) is a preferable treatment option as it helps in the intramedullary reduction of the radial head and therefore avoids potential damage to the proximal radial epiphysis and capsule [9,10].

The present study evaluates the functional results of Judet type III and IV radial neck fractures operated at a single center.

## Materials and methods

**Figure 1:**
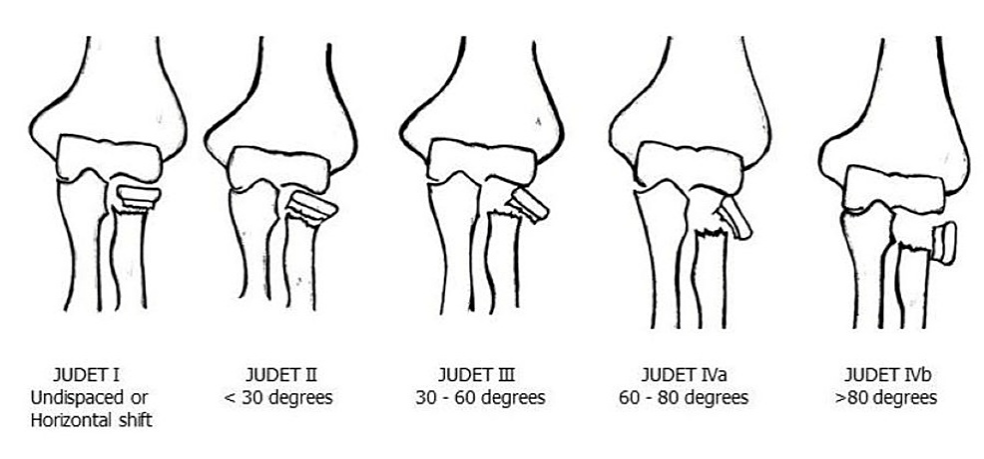
Judet classification of pediatric radial neck fractures

This was a retrospective study conducted by using medical records of nine patients who had displaced radial neck fractures treated at our institute which is a tertiary trauma care center between January 2012 and December 2021. Judet classification of pediatric radial neck fractures was followed in this series (Figure 1).

**Figure 2:**
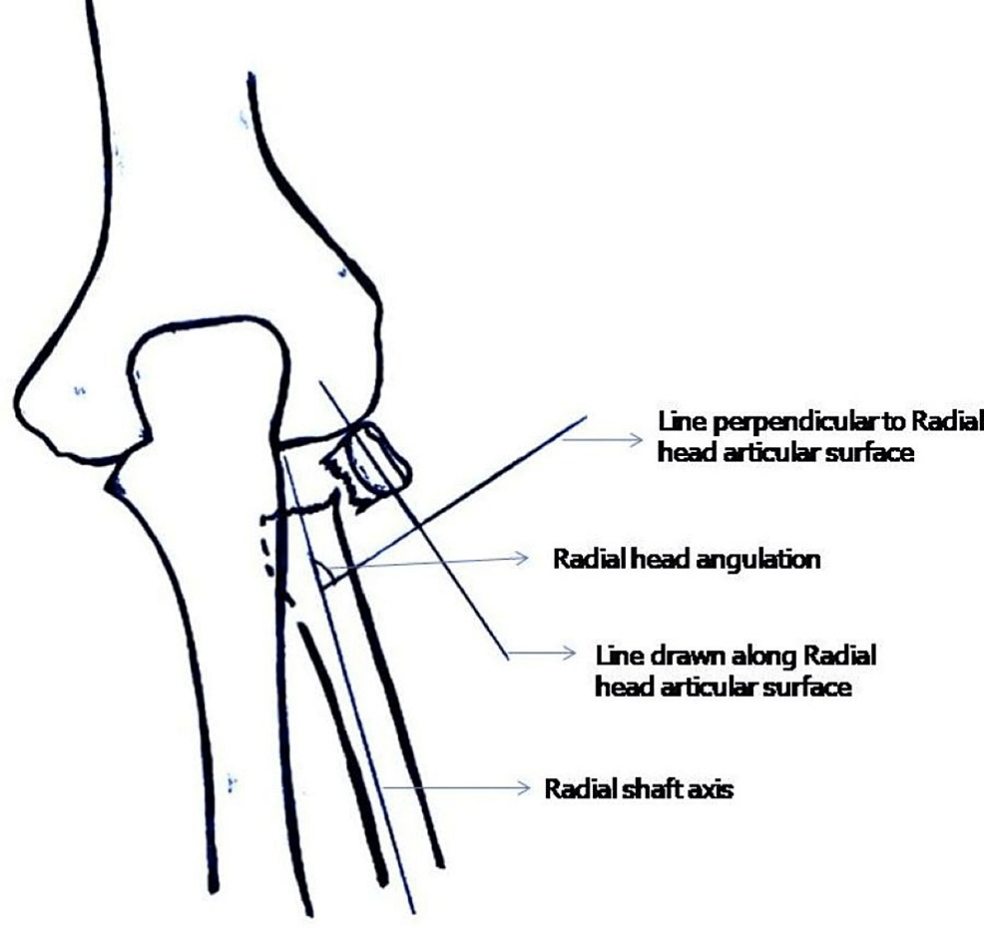
Measurement of radial head angulation

Radial head angulation is the angle measured between radial shaft axis and a line drawn perpendicular to articular surface of the radial head (Figure 2).

Judet type I is a non-displaced fracture, Judet type II involves angulation of less than 30°, and Judet type III involves angulation of between 30° and 60°. Type-IVa fractures are those with more than 60° of angulation of the radial head and type-IVb fractures are those with more than 80° of angulation of the radial head. Inclusion criteria were Judet type III and type IV radial neck fractures, open physis of radial neck at the time of fracture, minimum follow up time of six months. Exclusion criteria were open fractures and Judet type I and type II radial neck fractures.

Surgical technique

All the patients with elbow injuries were initially evaluated with anteroposterior, lateral, and oblique views and once the diagnosis of radial neck fracture was confirmed, limb was supported with an above-elbow posterior slab and were planned for surgery as early as possible.

**Figure 3:**
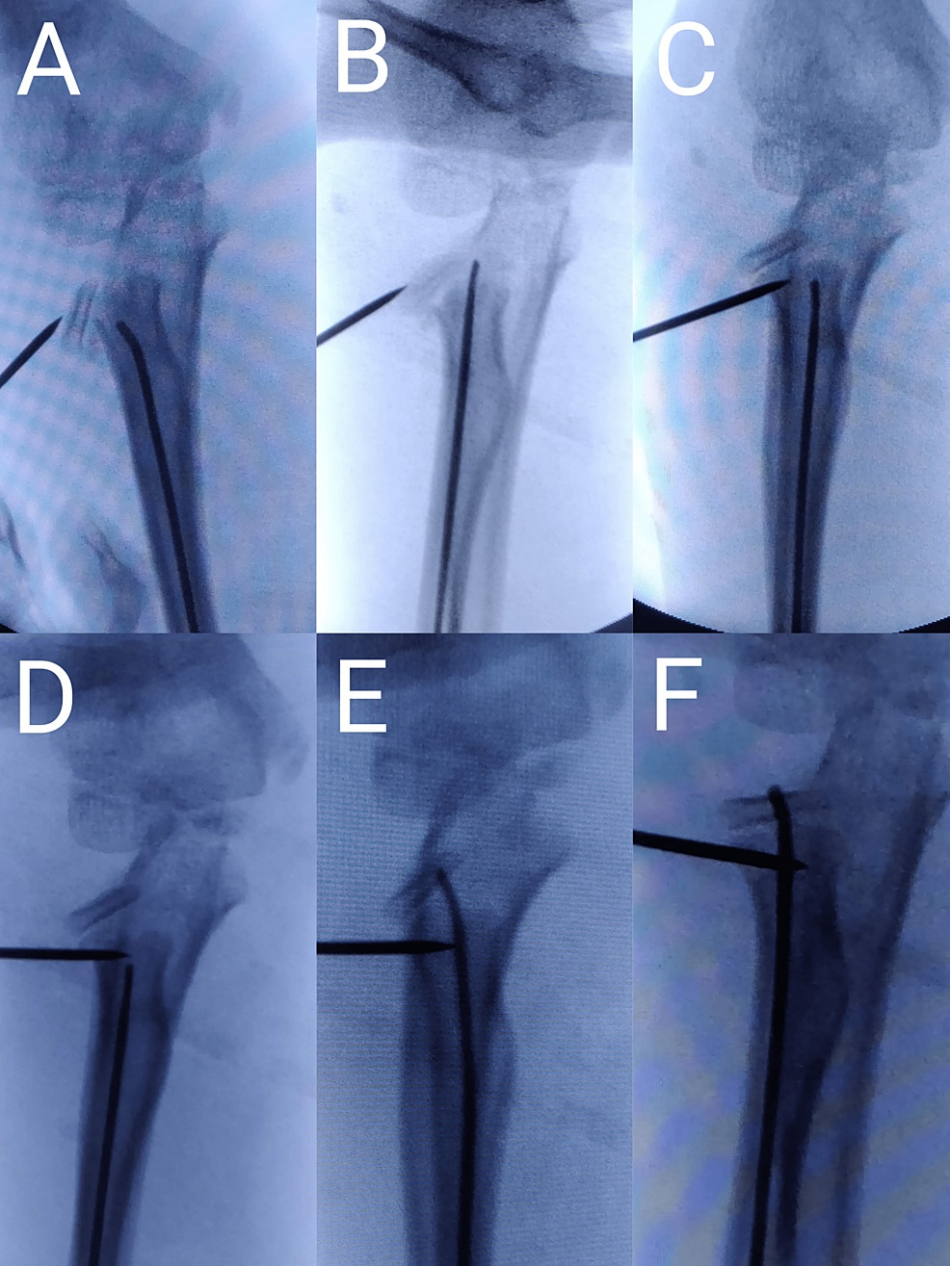
Pictures A to F show percutaneous joystick reduction of fracture followed intramedullary nailing by Metaizeau technique.

Under general anesthesia, the patient was kept in supine position and the involved upper limb was prepared and placed on a hand table. Initially, closed reduction was tried by Patterson’s method i.e., pulling the extended elbow in a varus direction and applying pressure on the radial head to reduce it. If reduction was not achieved, percutaneous K-wire joystick technique was used by checking under live fluoroscopic control in anteroposterior, lateral and oblique views to find the position of maximum displacement and angulation. If the satisfactory reduction was achieved with joystick, then the fracture was fixed with K-wire by passing K-wire from proximal radial epiphysis to metaphysis. In case of incomplete reduction, further reduction and maintainence of reduction was done with the Metaizeau technique. Only in one of our earlier cases (case 3) we resorted to transcapitellar K wire, which is highly discouraged by many authors in the literature. After Judet type IV was converted to Judet type III fracture with joystick technique, further reduction was obtained by Metaizeau technique of retrograde intramedullary nailing (Figure 3).

The nail not only helps in getting the reduction but also maintaining it. If reduction was not obtained with either of the techniques mentioned above, then open reduction was done by Kocher approach and K-wire fixation was done with forearm in pronation to prevent posterior interosseous nerve injury. Also, care was taken to preserve the periosteal hinge to decrease the chances of avascular necrosis of radial head. The K-wire removal was done at three to four weeks following which the gradual mobilization of the elbow started.

Patients were assessed for functional outcomes by the Mayo elbow performance score (MEPS), the Tibone and Stoltz functional criteria, and for complications at one year [14,15]. The MEP is based on four variables: pain, range of motion, stability and daily function [11]. Each of these variables were given certain points and all variables put together gives a sum of 100 points (Table 1).

**Table 1:** The Mayo elbow performance score (MEPS)

Variable	Definition	Points
Pain (maximum 45 points)	None	45
	Mild	30
	Moderate	15
	Severe	0
Range of motion, degrees (Maximum 20 points)	Arc > 100	20
	Arc 50-100	15
	Arc < 50	5
Stability (Maximum 10 points)	Stable	10
	Moderately unstable	5
	Grossly unstable	0
Function (Maximum 25 points)	Comb hair	5
	Feed oneself	5
	Personal hygiene	5
	Put on shirt	5
	Put on shoe	5

The results were categorized into four groups based on total points obtained: excellent >90 points; good 75 to 89 points; fair 60 to 74 points; poor <60 points. The Tibone and Stoltz classification for functional results is based on pain, deformity, and range of motion and is shown in Table 2.

**Table 2:** Tibone and Stoltz classification for functional results of radial neck fractures ROM: Range of motion

Clinical condition of the patient	Result
Pain-Absent Deformity-Absent ROM-Complete	Excellent
Pain-Occasional Deformity- Carrying angle increase <10 degrees ROM-Limited < 20 degrees	Good
Pain-Occasional Deformity- Carrying angle increase >10 degrees ROM-Limited >20 degrees	Fair
Radial head removal required to treat pain or ROM limitation	Poor

## Results

**Figure 4:**
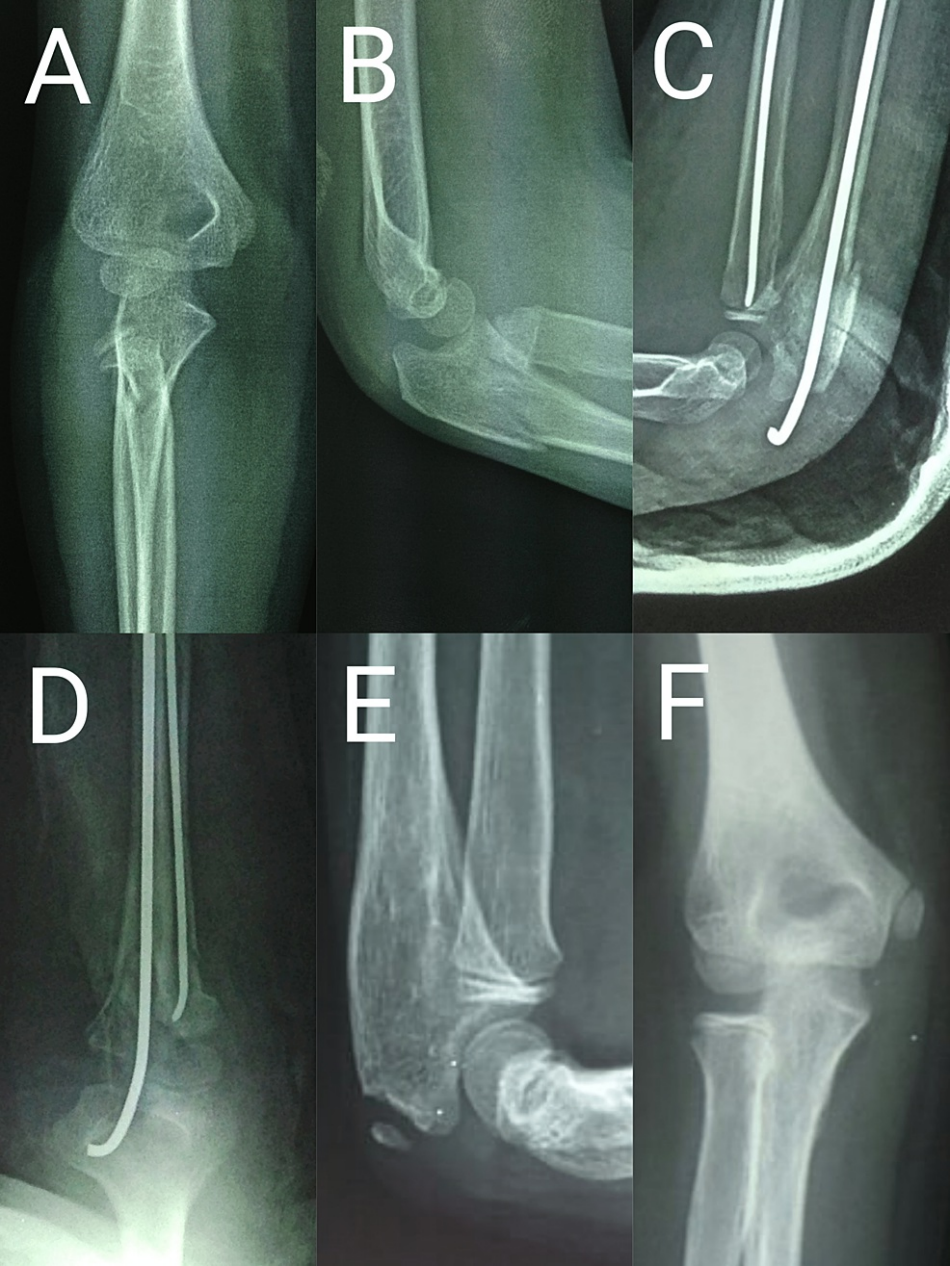
Radiographs of a nine-year-old male patient A and B: Preoperative radiograph of nine-year-old male showing Judet type III radial neck fracture and proximal ulna fracture; C and D: Immediate postoperative radiograph showing intramedullary nailing of radial neck fracture and K-wire fixation of ulna; E and F: Radiograph at the two-year follow-up showing fracture union

**Figure 5:**
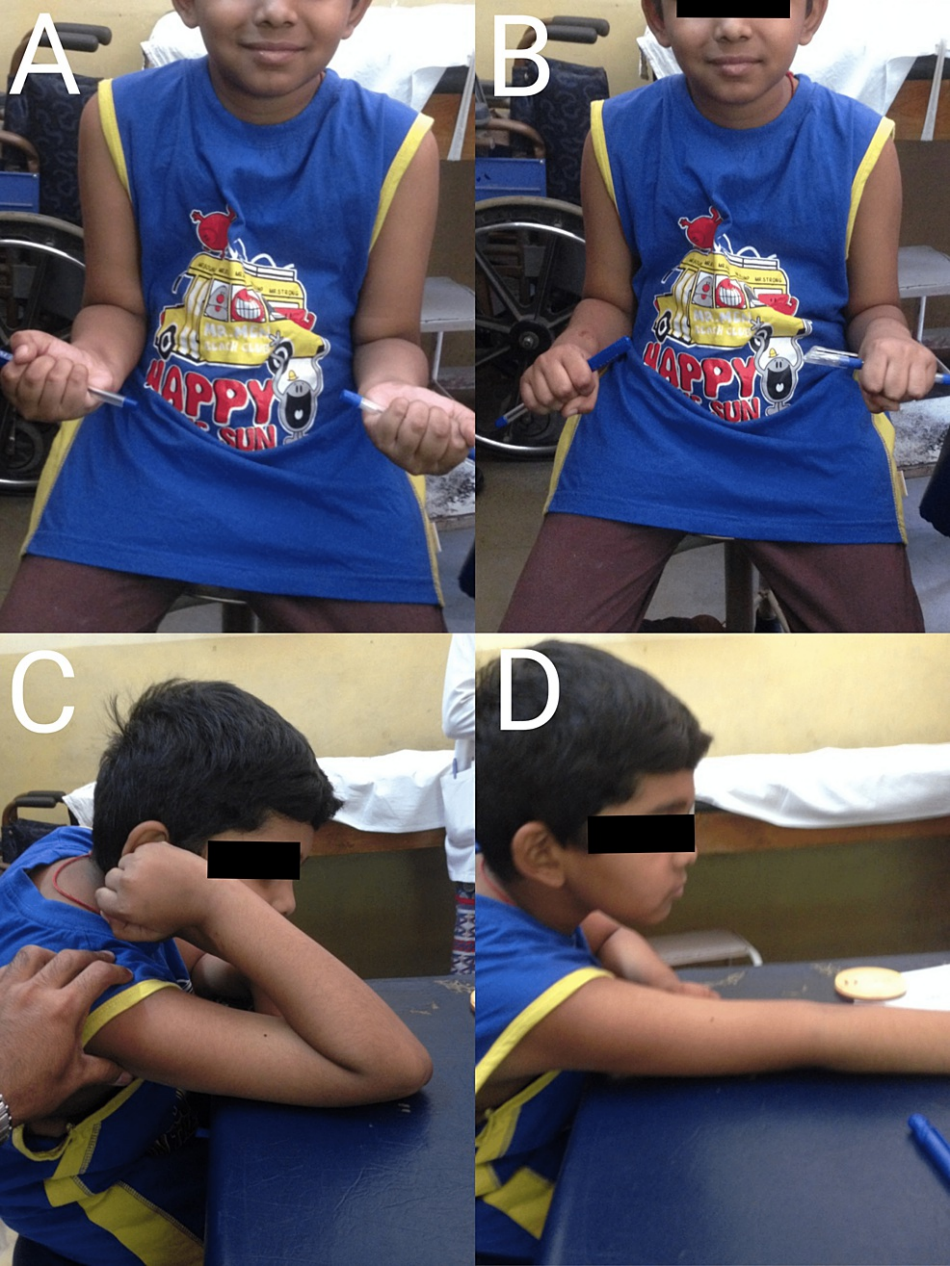
Pictures A to D show excellent function after closed reduction and intramedullary nailing by the Metaizeau technique of the male patient.

**Figure 6:**
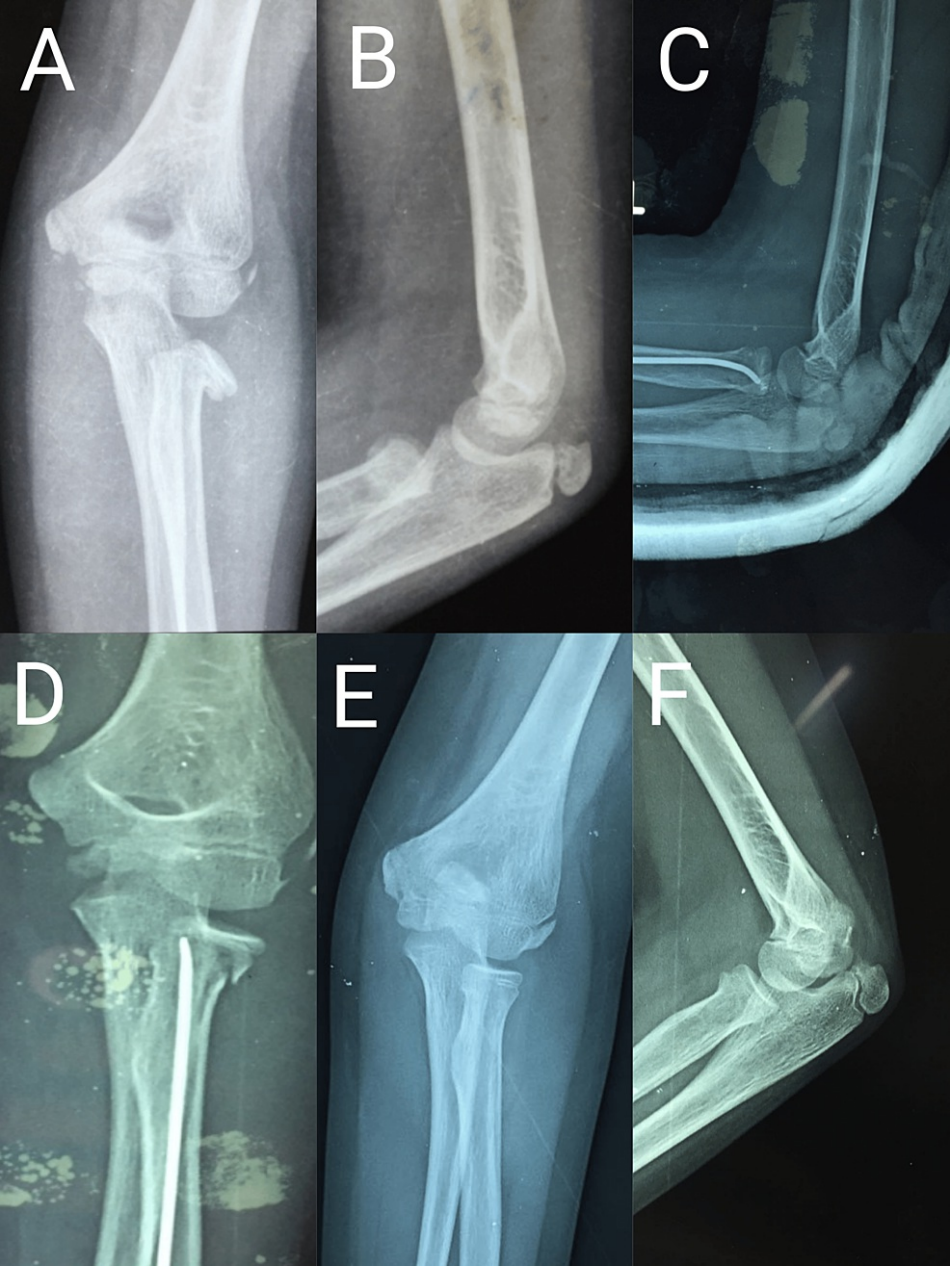
Radiographs of a nine-year-old female patient A and B: Preoperative radiograph of a nine-year-old female patient showing Judet type IV radial neck fracture; C and D: Immediate postoperative radiograph after percutaneous K-wire assisted joystick reduction followed by intramedullary nailing by the Metaizeau technique; E and F: Radiograph at the two-year follow-up showing fracture union

**Figure 7:**
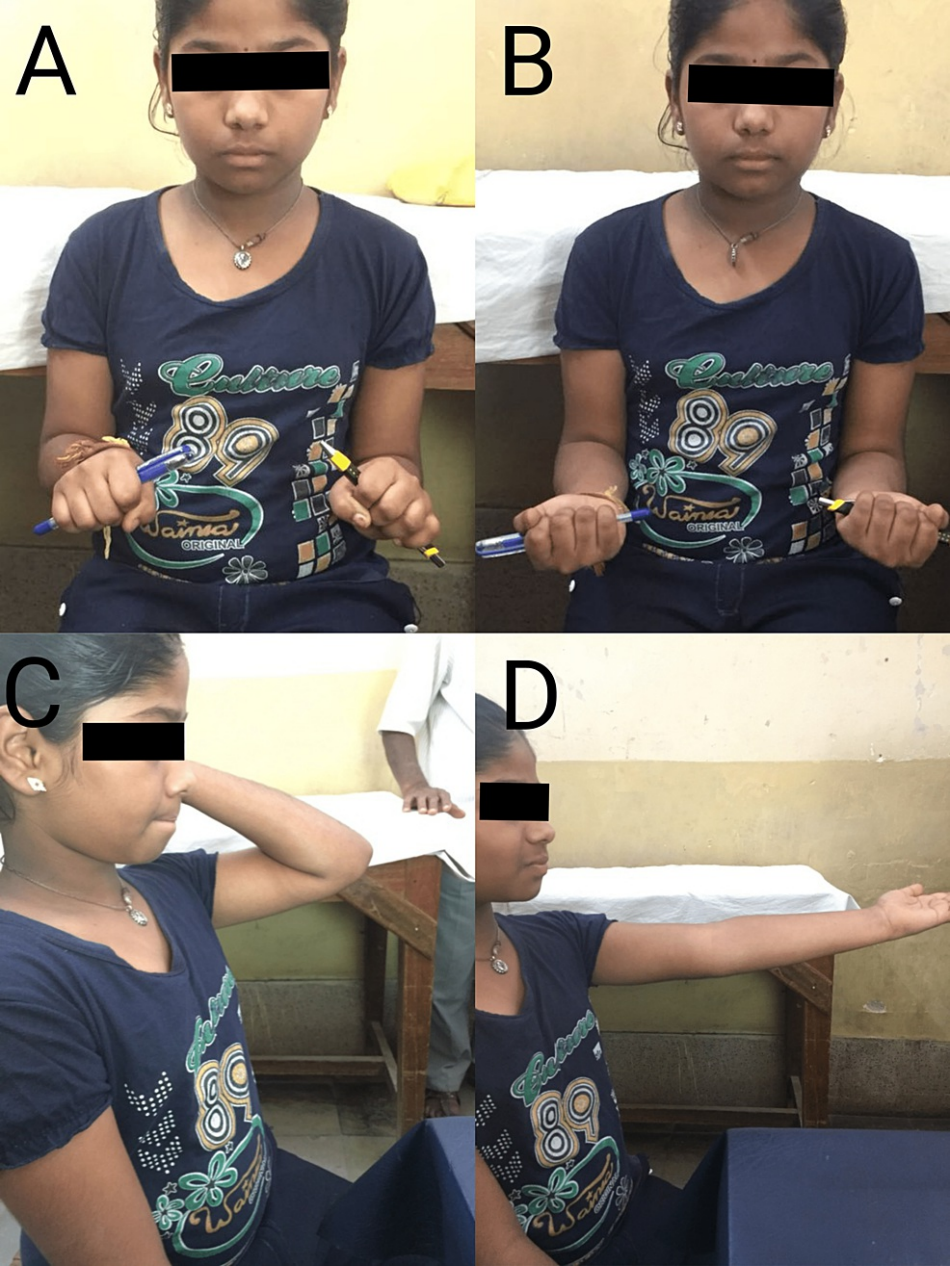
Pictures A to D show excellent function after percutaneous K-wire assisted joystick reduction followed by intramedullary nailing by the Metaizeau technique of the female patient.

The mean age of the patients was 9.14 ± 2.2 years (range: four to 11 years). Seven(77.8%) patients were males and two (22.2%) patients were females. The right side was the most commonly injured side (right at 67% and left at 33%). Five (55%) cases were of Judet type III and IV (45%) cases were of Judet type IV. The mean fracture angulation of the series was 56.5 degrees (range 33.2 degrees to 79.2 degrees). Four patients had isolated radial neck fractures and four patients had associated proximal ulna fractures and one patient had associated posterolateral elbow dislocation. Closed reduction and intramedullary nailing by Metaizeau technique were done in three cases and percutaneous joystick reduction and intramedullary nailing by Metaizeau were done in two cases (Figures 4-7).

Four cases required open reduction and K-wire fixation as closed and percutaneous pin leverage techniques did not achieve acceptable reduction. The mean follow-up of all patients was four years (range: six months to seven years and five months). Three cases treated with closed reduction and intramedullary nailing by the Metaizeau technique had excellent functional results. Among two patients treated with percutaneous pin leverage and intramedullary nailing by the Metaizeau technique, one patient had an excellent outcome and the other a good outcome. Among four cases treated with open reduction and K-wire fixation, two patients had good outcomes, one patient had fair outcomes, and one patient had a poor outcome. The poor outcome in one patient (case 8) was due to associated posterolateral dislocation of the elbow and loss of elbow flexion-extension and pronation-supination. The other case with a fair result, we believe, was the one where we used a transcapitellar wire. Another complication we encountered was posterior interosseous nerve palsy in two cases (case 1 and case 6) which recovered in four to six weeks. A summary of all nine cases is shown in Table 3.

**Table 3:** Summary of the nine cases CR: Closed reduction,  OR: Open reduction

S.NO.	Age (years)	Sex	Judet type	Side of injury	Associated injuries	Treatment	Follow up	MEPS	Tibone and Stoltz classification	Complications
1	9	M	3	Right	Ulna fracture	CR+Metaizeau	4 years 5 months	Excellent	Excellent	Posterior interosseous nerve palsy
2	11	M	3	Left	Ulna fracture	OR + K wire	7 years 5 months	Good	Good	None
3	4	F	4	Left	None	OR+ transcapitellar wire	6 years 4 months	Fair	Fair	Stiffness
4	9	M	3	Right	Ulna fracture	CR + Metaizeau	4 years 11 months	Excellent	Excellent	None
5	9	F	4	Left	None	Percutaneous pin leverage +Metaizeau	3 years 10 months	Excellent	Excellent	None
6	11	M	4	Right	Ulna fracture	Percutaneous Pin leverage+ Metaizeau	3 years 9 months	Good	Good	Posterior interosseous nerve palsy
7	11	M	4	Right	None	OR and K-wire fixation	4 years 1month	Good	Good	None
8	10	M	3	Right	Posterolateral elbow dislocation	CR of dislocation + OR and K-wire fixation	6 months	Poor	Poor	Stiffness
9	8	M	3	Right	None	CR+Metaizeau	13 months	Excellent	Excellent	None

## Discussion

Radial neck fractures are relatively rare fractures in the pediatric age group and management of displaced radial neck fractures is challenging. The Judet type I and type II fractures are managed nonoperatively and they heal with acceptable functional results [5]. The Judet type III and type IV radial neck fractures are managed surgically and various surgical options like percutaneous pin leverage method, elastic stable intramedullary nailing, and open reduction with or without internal fixation are available [7-12].

The mean age in the present study was 9.1 years. This is closer to the operative group of a study by De Mattos et al. [16]. The mean fracture angulation in this series was 56.5 degrees (range 33.2 degrees to 79.2 degrees) and was comparable to the study by Klitscher D et al. (62 degrees) [17]. Approximately 30% to 50% of patients with radial neck fractures have associated injuries like fractures of the proximal ulna, medial and lateral epicondyle, ruptures of the medial collateral ligament, elbow dislocation, etc. [15,18]. In this series, four patients had associated proximal ulna fractures and one patient had associated posterolateral dislocation of the elbow. The proximal ulna was treated with closed reduction and intramedullary K-wire fixation in all cases and elbow dislocation was reduced by closed manipulation under general anesthesia. It is reported that associated fractures negatively influence the functional outcome of radial neck fractures [15,19]. In our series, among five patients with associated fractures, two patients had excellent results, two patients had good results, and one patient had poor results.

Closed reduction and intramedullary nailing by the Metaizeau technique were done in three cases. All these cases were Judet type III fractures and had excellent functional results as per the MEPS and the Tibone and Stoltz classification. Our results were in agreement with a study done by Metaizeau et al. and Klitscher D et al. in which they report excellent results in 100% of Judet type III fractures treated using closed intramedullary nailing [9,17]. This can be attributed to the ease of reduction of Judet type III fractures as they rarely require percutaneous methods of reduction or open reduction. So, there are minimal chances of additional injury to the radial head and surrounding soft tissues.

Two cases were treated by percutaneous joystick reduction with K-wire followed by intramedullary nailing. The reduction was assessed by looking for radiocapitellar alignment in lateral view under C-arm and by doing intraoperative pronation and supination. If these movements were satisfactory, then the reduction was considered adequate. Among these, one case was Judet type III and the other one was Judet type IV fracture. One case had an excellent functional result and the other had good functional results according to MEPS, and Tibone and Stoltz classification. Brandão et al. and D’Souza et al. concluded that the percutaneous reduction with K-wire followed by intramedullary fixation with the Metaizeau technique gives better functional results compared to open reduction in displaced radial neck fractures [20,21]. Similarly, Klitscher et al. reported excellent results in all patients who underwent percutaneous K-wire reduction and intramedullary nailing [17]. These functional results with percutaneous joystick reduction can be attributed to the prevention of further injury to soft tissues and blood supply of the radial head, avoiding fibrous capsular adhesions that occur with an open reduction which severely restrict the range of motion.

Despite all techniques of closed and percutaneous reduction, at times acceptable reduction may not be achieved where open reduction becomes the only option. In this series, four patients were operated on with open reduction and K-wire fixation. Two patients had good results, one patient had a fair result, and one patient had a poor result. The poor result in one case (case 8) may be due to the associated unreduced posterolateral elbow dislocation and delayed presentation (12 days after injury following initial treatment by a quack) which might have severe soft tissue and capsular injury. Several studies have reported that open reduction will give poor functional outcomes despite the anatomical reduction of the fracture [22-24]. These poor results may be due to the associated complications with an open reduction such as avascular necrosis, proximal synostosis, heterotopic ossification, infection, premature physeal closure, and loss of range of motion due to fibrous adhesions. Kaiser et al. studied 19 severely displaced radial neck fractures (Judet IV) comparing those with and without bony contact and concluded that the unfavorable results are because of the nature of the initial injury and not because of open reduction [25]. However, the authors in the present study cannot make such inferences as this is a small case series.

Shah et al. described a new technique of closed reduction to obtain an anatomical and stable reduction in Judet type III and IV fractures [26]. This technique involves the application of varus stress at the elbow with thumb pressure applied at the radial head at the posterolateral aspect like in Patterson’s technique, after which the elbow is hyperflexed and hyperpronated simultaneously with continuous pressure over the radial head. The arm is immobilized in an above-elbow cast in 90-degree flexion and mid-prone position, with no K-wire or nail fixation after achieving complete reduction. The authors achieved excellent results without complications in all patients in their series.

Elbow stiffness, radio-ulnar synostosis, avascular necrosis, posterior interosseous nerve injury, heterotopic ossification, nonunion, and malunion are some of the known complications of radial neck fractures with open or closed methods of treatment [13]. Elbow stiffness and posterior interosseous nerve palsy were the complications encountered in our series. The loss of range of movement was more in the pronation-supination plane than in flexion-extension and was seen in cases treated with open reduction. We attribute this loss of range of motion to fibrous adhesions formed in the soft tissues after open reduction.

All the reported cases here were from a single institute and were operated by the experienced senior author SKC. Our study is not without limitations. The main drawbacks are the retrospective nature of the study, small sample size, and varied modalities of treatment. Another drawback is the short follow-up, so we could not assess long-term complications like avascular necrosis of the radial head, cubitus valgus, etc. The authors are of the opinion that the small sample size is justified owing to the rarity or lesser incidence of this fracture. The authors would like to try the recently published Shah et al.’s technique of closed reduction in severely displaced fractures without the need for internal fixation in their future cases and compare the results with the present patient database [26].

## Conclusions

Majority of moderately to severely displaced pediatric radial neck fractures which need intervention can be managed by the closed reduction technique of Metaizeau with or without pin leverage with excellent to good functional outcomes at short-term follow-up. Some cases need open reduction which also has good to fair outcomes. Initial trauma and associated injuries seem to play a role in the outcome rather than the treatment method per se. However, a larger sample size and longer follow-up are needed for comparisons and for arriving at better and definitive conclusions.
